# Libby amphibole‐induced mesothelial cell autoantibodies bind to surface plasminogen and alter collagen matrix remodeling

**DOI:** 10.14814/phy2.12881

**Published:** 2016-08-12

**Authors:** Robert Hanson, Caryn Evilia, John Gilmer, Linda Woods, Brad Black, Raja Flores, Jean C. Pfau

**Affiliations:** ^1^ Department of Biological SciencesIdaho State UniversityPocatelloIdaho; ^2^ Department of ChemistryIdaho State UniversityPocatelloIdaho; ^3^ Center for Asbestos Related DiseasesLibbyMontana; ^4^ Department of Thoracic SurgeryIcahn School of Medicine at Mt SinaiNew York CityNew York; ^5^ Present address: Brigham Young UniversityProvoUtah; ^6^ Present address: Montana State UniversityBozemanMontana

**Keywords:** Asbestos, autoimmunity, collagen, Libby amphibole, plasminogen, pleural fibrosis, proteomics

## Abstract

Lamellar pleural thickening (LPT) is a fibrotic disease induced by exposure to Libby amphibole (LA) asbestos that causes widespread scarring around the lung, resulting in deterioration of pulmonary function. Investigating the effects of autoantibodies to mesothelial cells (MCAA) present in the study populations has been a major part of the effort to understand the mechanism of pathogenesis. It has been shown in vitro that human mesothelial cells (Met5a) exposed to MCAA increase collagen deposition into the extracellular matrix (ECM). In this study, we sought to further elucidate how MCAA drive increased collagen deposition by identifying the protein targets bound by MCAA on the cellular surface using biotinylation to label and isolate surface proteins. Isolated surface protein fractions were identified as containing MCAA targets using ELISA. The fractions that demonstrated binding by MCAA were then analyzed by tandem mass spectrometry (MS/MS) and MASCOT analysis. The most promising result from the MASCOT analysis, plasminogen (PLG), was tested for MCAA binding using purified human PLG in an ELISA. We report that serum containing MCAA bound at an optical density (OD) 3 times greater than that of controls, and LA‐exposed subjects had a high frequency of positive tests for anti‐PLG autoantibodies. This work implicates the involvement of the plasminogen/plasmin system in the mechanism of excess collagen deposition in Met5a cells exposed to MCAA. Elucidating this mechanism could contribute to the understanding of LPT.

## Introduction

Lamellar pleural thickening (LPT) is an emerging disease distinguished from other forms of pleural fibrosis because it results in a diffuse thickening of the pleural lining coupled with loss of pulmonary function (Black et al. 2014). Various forms of pleural fibrosis occur in individuals who suffer from pleural infections or other damage to the mesothelial lining, such as exposure to asbestos. LPT has been described primarily among individuals exposed to asbestos, and garnered particular attention in the wake of exposures to Libby amphibole (LA) asbestos as a progressive disease with severe compromise in pulmonary function (Whitehouse 2004; Black et al. 2014). Evidence linking both occupational and environmental exposure to LA with increased mortality and morbidity has been reported (Alexander et al. 2012; Antao et al. 2012; Black et al. 2014; Lockey et al. 2015). In the amphibole‐exposed population of Libby MT, where exposures occurred due to the mining of asbestos‐contaminated vermiculite, pleural fibrosis is the predominant cause of morbidity and mortality (Peipins et al. 2003; Larson et al. 2012). Within the health screening population of the Center for Asbestos Related Diseases (CARD) clinic in Libby, 117 of 203 documented deaths (57%) were attributed to asbestos‐related diseases, and of those, 83 (71%) presented with LPT (B. Black, personal communication). A recent study of CT scans from workers exposed to low levels of LA at a manufacturing plant in Marysville, OH, reported that 53% had pleural thickening and 13% showed parenchymal changes (Lockey et al. 2015). Like other forms of pleural fibrosis, LPT is refractory to treatment, leaving patients and physicians without many options to prevent respiratory decline. In view of the discovery of extensive exposures to similar amphiboles in the southwestern U.S. (Buck et al. 2013; Metcalf and Buck 2015), many new cases of LPT may appear, necessitating new treatment modalities.

Previous studies have shown correlations between the presence of mesothelial cell autoantibodies (MCAA) and the existence of conditions that could lead to the development of LPT (Marchand et al. 2012; Serve et al. 2013). Specifically, among a subset of Libby subjects, the presence of MCAA in serum was associated specifically with pleural, but not interstitial, disease (Marchand et al. 2012). In vitro, exposure to MCAA increased collagen matrix formation by cultured human mesothelial cells (Serve et al. 2013). Because these studies implicate MCAA in pleural fibrosis, the first step toward validation of that hypothesis required identification of potential candidate targets for those antibodies. Tandem mass spectrometry (MS/MS) can be used for both exploratory and hypothesis‐directed proteomics. This is due largely to the bioinformatics tools available to analyze the peptide sequences identified by the method (Perkins et al. 1999; McHugh and Arthur 2008).

In this study, we sought to identify the antigen(s) bound by MCAA on the surface of human mesothelial cells. We proposed to identify potential candidates by screening for MCAA binding in fractions of surface proteins that had been isolated by affinity capture and size exclusion chromatography. Those with high affinity for MCCA would be analyzed via MS/MS, particularly looking for any protein(s) identified in more than one fraction. We followed up on the most promising candidate by testing for binding by MCAA in serum from LA‐exposed subjects, and demonstrating its role in altered collagen matrix deposition.

## Materials and Methods

### Human serum samples

Human serum samples from LA‐exposed subjects were obtained from the CARD clinic in Libby Montana, and stored at −80°C until use, as described in Marchand et al. (2012). Idaho State University IRB project approval permitted collection, transfer, and testing of the samples in our laboratory. Samples previously identified as MCAA positive (MCAA+) or negative (MCAA−) by cell‐based ELISA (Marchand et al. 2012) were pooled and small aliquots stored at −20°C. Pooled sera cleared of IgG antibodies were used as negative controls. Samples were cleared of IgG using Protein G beads. Removal of detectable IgG was confirmed by comparing cleared sera to uncleared sera via SDS‐PAGE stained with Coomassie Blue. Normal human serum samples were obtained from Precision Med (Solana Beach, CA), in order to test for antibodies to (PLG) in sera from the general population. Age and sex demographic data were provided by the company, and this set of samples was matched to the MCAA+ and MCAA− sera for age and sex (Table 1).

**Table 1:** Demographics data for human serum samples

	MCAA+	MCAA−	NHS	PLG+
Age (mean)	61.0	59.2	58.7	59.6
Age (SD)	10.5	13.3	3.7	12.3
M:F (ratio)	33:23 (1.43)	30: 21 (1.43)	25: 15 (1.67)	27: 18 (1.5)

NHS, normal human serum.

### Cell culture

Nonmalignant, transformed human mesothelial cells, Met‐5A (ATCC, CRL9444, Manassas, VA) were grown and maintained in tissue culture flasks, using RPMI medium (CellGro Mediatec, Manassas, VA) supplemented with 5% fetal bovine serum (FBS; Atlanta Biologics, Lawrenceville, GA) and 1% Penicillin/Streptomycin (CellGro). Cells were maintained at 37°C and 5% CO_2_ in a humidified cell culture incubator (Thermo Scientific, Rockford, IL).

### Biotin labeling and affinity chromatography

Met5A cells were grown in T‐75 flasks until ~85% confluency was reached. The cells were rinsed with two washes of ice‐cold PBS for no more than 5 sec each wash. The cells were then covered with Pierce sulfo‐biotin labeling solution (Pierce Cell Surface Protein Isolation Kit; Life Technologies, Grand Island, NY) and agitated for 30 min at 4°C. The reaction was stopped by adding 500 *μ*L of quenching solution to each flask. The cells were then scraped into suspension and centrifuged at 500 *g* for 3 min. The flasks were rinsed with 10 mL 25 mmol/L Tris Buffered Saline (TBS) and the cells removed were added to the total cell pellet.

The cells were lysed for 30 min at 4°C using the kit's lysate buffer containing the protease inhibitor cocktail (Pierce, Thermo Fisher, Waltham, MA) and sonicated five times for 1 sec each at 40 kHz (Branson Ultrasonic, Danbury, CT). The lysate was placed in an ice bath for 30 min and was sonicated every 5 min for 1 sec to improve solubility. The lysate was then clarified by centrifugation at 10,000 *g* for 2 min. The supernatant was transferred to a fresh tube.

The lysate was then added to a freshly washed neutravidin agarose affinity column. This was incubated at 25°C under constant rotation for 60 min. The column was washed using the kit's wash buffer treated with a protease inhibitor cocktail (Pierce). The column was then washed three times by adding protease inhibitor‐treated wash buffer and centrifuging for 2 min at 1000 *g*. The protein was eluted from the column by reducing the disulfide bond in the biotin tag using 50 mmol/L DTT at 25°C for 60 min. Protease inhibitors were then added to the eluate. The lysate was kept at 4°C until it was added to the size exclusion column.

### Size exclusion chromatography

Surface protein lysate/suspension (1 mL) was fractionated using a GE Healthcare AKTA Purifier FPLC system and a 16/60 Sephacryl S‐100 HR size exclusion column equilibrated in PBS. Isocratic elution of the fractionated proteins was achieved, using PBS buffer at a rate of 0.5 mL/min. 1.5 mL fractions were collected in autoclaved test tubes and transferred to 1.5 mL centrifuge tubes on ice within 15 min of eluting from the column. The total number of fractions collected was 143.

### BCA analysis of fractions

Protein concentration in the fractions was determined using a Pierce BCA kit. The screening of fractions 1 through 70 was optimized to detect between 5 and 250 *μ*g as directed by the kit protocol. The rest of the samples were processed using the standard protocol.

### Protein lysate MCAA‐binding ELISA

A quantity of 200 ng of protein from each sample, as determined by BCA assay (Pierce), was placed in 100 *μ*L of carbonate coating buffer in wells of a polystyrene high affinity 96 well plate and left overnight at 4°C. The buffer was washed from the plate using 200 *μ*L of PBS per well. This was repeated three times. The plates were then blocked with 200 *μ*L of 5% nonfat dry milk in PBS for 1 h at 25°C. The blocker was then aspirated off twice and the plates were incubated with 100 *μ*L buffer‐containing MCAA positive serum diluted at a ratio of 1/1000 in 3% BSA overnight at 4°C. The next day, the plates were washed three times with PBS, waiting 4 min between washes. The plates were then incubated with goat anti‐human IgG HRP for 1 h at 37°C. The plates were washed as above. The plates were developed using 100 *μ*L of Pierce one‐step TMB ELISA substrate per well. Development was stopped by adding 50 *μ*L of 1 mol/L HCl. Plates were analyzed at 450 nm on a microtiter plate reader (BioTek Instruments, Winooski, VT). Correction for nonspecific secondary antibody binding was done on a plate‐to‐plate basis by subtracting the mean OD for the secondary antibody‐only control wells from the mean OD of each sample.

### Sample preparation for tandem MS: in solution tryptic digestion

A quantity of 5 *μ*L of 1 mol/L ammonium bicarbonate (pH 8.0) was added to 45 *μ*L of the protein suspension. Then, 5.5 *μ*L of 10 mmol/L DTT was added this solution and the reaction was placed in a heat block set at 50°C for 15 min. The reaction was cooled to room temperature. Then, 13.9 *μ*L of iodoacetamide was added to each reaction, and the solution was allowed to react at room temperature in the dark for 20 min. Proteomics grade Trypsin (Sigma Aldrich, St. Louis, MO) was added at a 1:100 dilution. The samples were incubated at 37°for 16–20 h. Acetic acid was added to acidify and stop the trypsin digestion.

### Desalting and shipping

Samples were desalted using a C18 ziptip (Millipore, Billerica, MA) according to the manufacturer's instructions. The eluted sample was then placed in a SpeedVac and evaporated to dryness. Samples were then shipped to the Mass Spectrometry CORE facility at the University of Idaho, Moscow, ID.

### Tandem MS analysis

**Figure 1:**
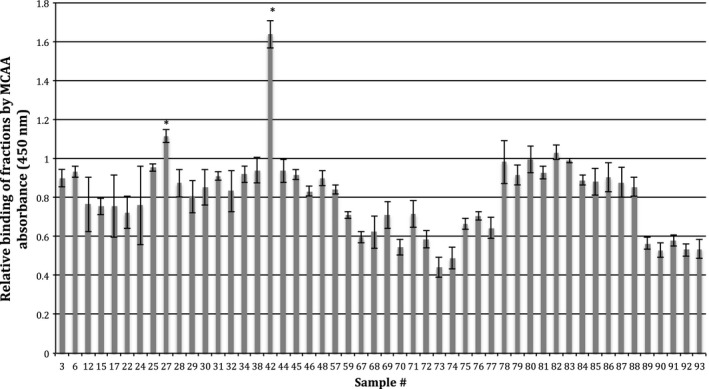
Protein fraction ELISA for MCAA binding. The fractions with sufficient protein were then analyzed using an ELISA protocol as described in the Materials and Methods. Samples were prepared in triplicate and analyzed by absorbance on a plate reader set to 450 nm. Standard error of the mean was calculated; and samples were considered of interest if they had an absorbance significantly higher than sample 78, which had a mean absorbance of 1 (*P < 0.01, *n* = 3 wells per fraction) by unpaired two‐tailed *t*‐test. This graph is representative of three separate experiments.

The MS/MS spectra were obtained from an in‐solution digestion of fractions 27, 42, and neighboring fraction 30 (Fig. 1). Analysis of peptides was done using reverse phase liquid chromatography on a Waters nanoAcquity Ultra performance liquid chromatograph (UPLC). The analytical column used was a 100 *μ*m (i.d.) × 100 mm (l) BEH 130 C18 nanoAcquity UPLC column (Waters Corp. part # 186003546). The solvents used were 0.1% formic acid in water (solvent A) and 0.1% formic acid in acetonitrile (solvent B). The initial solvent mixture was 95% A and 5% B and the flow rate was 0.4 *μ*L/min. Starting 1 min after injection, the concentration of solvent B was increased to 15% over the next 10 min. The concentration of B was then increased to 27% over the next 10 min, increased to 41% over the next 10 min, increased to 57% over the next 10 min, and finally increased to 90% over the next 14 min. The mobile phase was held at 90% B for the next 5 min, and then returned to 5% B over the next 10 min. The sample volume injected was 2 *μ*L and was loaded onto a 180 *μ*m × 20 mm Symmetry C18 (5 *μ*m) trap column (Waters Corp., part # 186002841) prior to injection onto the analytical column. The trapping conditions were a flow rate of 8 *μ*L/min of 100% solvent A for a duration of 3 min.

The MS/MS spectra of peptides suitable for protein database matching were generated using a Waters Micromass Q‐Tof Premier quadrupole‐time of flight mass spectrometer using a nanospray electrospray ionization inlet. The nanosprayer was fitted with a 20 *μ*m (i.d.) fused silica emitter tip. The UPLC and the Q‐Tof MS were controlled, using MassLynx V4.1 software (Waters Corp., Milford, MA). Data generation was done using a data directed analysis (DDA) MS method. This method allows the LC effluent to be monitored in MS survey mode. When multiple charged analytes (+2, +3, and +4) with properties characteristic of peptides were detected, the instrument switched to MS/MS mode, the analyte precursor m/z was selected, and the collision cell voltage was increased to produce peptide fragmentation MS/MS spectra suitable for sequencing. The conditions for the MS survey were a mass range setting of 300–2000 Da and a scan time of 1 sec/scan. MS/MS acquisition was limited to those multiple positively charged ions producing a signal over 15 cps. The MS/MS spectra were recorded over the range of 50–2000 Da at a scan rate of 2 sec/scan. The collision voltage was ramped from 15 to 45 V during MS/MS spectrum acquisition. The mass spectrometer tune page settings used were—capillary voltage was 3.65 kv, cone voltage was 42 V, source temperature was 110°C, sheath gas pressure was 0.3 bar, the collision energy was 5 V, and the detector voltage was 2150 v. A reference compound, or lock mass, was sprayed simultaneously with the LC effluent from a lock mass sprayer and was sampled for 1 sec every 30 sec. The lock mass standard used was human [glu^1^]‐fibrinopeptide B (Sigma cat. # F‐3261), which produces a doubly charged peak at m/z of 785.8426 which was used for mass correction. The raw data were processed using Protein Lynx Global Server (PLGS) V2.3 software (Waters Corp.). Data searches were done using a SwissProt database in PLGS and the NCBI nonredundant database in Mascot (Matrix Science, Boston, MA).

### Bioinformatics searches

The STRING 10 protein interaction network was selected to analyze potential connections to ECM deposition or degradation because it provides weighted connections within the generated networks (Szklarczyk et al. 2015). Searches were performed using the specific names of candidate genes, e.g., COL1A1, and the key words ‘collagenase’, ‘collagen’. All return hits were included in the network. The network was then reduced to only those proteins that had a connection to one of the protein candidates and COL1A1.

### Cell‐based ELISA for detecting MCAA binding in serum

A cell‐based ELISA was used as previously described (Serve et al. 2013). In brief, Met5a cells were seeded at 100,000 cells per well in a 96 well plate. The cells were allowed to adhere overnight at 37°C and then fixed in 1% paraformaldehyde. The plates were blocked for 1 h at room temperature in 5% nonfat dry milk in PBS. The cells were then exposed to either serum that had been identified as MCAA+ or MCAA+ that had been cleared of selected components; either total IgG or antibodies to target antigen (PLG). The serum was diluted in 3% BSA in PBS at a 1/1000 ratio. The cells were incubated at room temperature for 1 h and then washed for 4 min in 0.05% PBS Tween. This was repeated three times. The plate was then blocked a second time in 5% nonfat milk in PBS for 1 h. The wells were then incubated with 100 *μ*L of 3% BSA PBS containing a goat antihuman IgG HRP secondary antibody, diluted at 1/1000. The plate was again incubated under agitation for 1 h at room temperature. The plate was washed for 4 min in 0.05% PBS Tween and repeated three times. The plates were then developed with 100 *μ*L of Pierce One Step TMB ELISA reagent. The reactions were stopped with 50 *μ*L of 1 mol/L HCL. The plates were then read for absorbance at 450 nm on the BioTek plate reader.

### Detection of serum antibodies to plasminogen

Purified human PLG (R&D Systems, Minneapolis, MN) was coated to high binding ELISA plates in carbonate coating buffer (pH 8) overnight. After blocking with 5% nonfat dry milk for 2 h, samples from LA‐exposed MCAA positive and negative serum samples were added to wells in duplicate. Before being added to the wells, the serum was diluted 1:100 in 3% BSA in PBS. Serum samples from healthy controls that had been shown to be MCAA negative by cell‐based ELISA as previously described (Marchand et al. 2012) were used to provide a control group. After 2 h at room temperature, the wells were washed in PBS‐Tween and then stained with anti‐human IgG HRP antibodies for 1 h, followed by development, using TMB substrate. The optical density of the wells was analyzed at 450 nm using the BioTek plate reader. Samples were determined to be anti‐PLG positive if the absorbance value for that sample was at least three standard deviations above the mean absorbance for wells with known MCAA‐negative controls. The data are also reported for each serum sample as the number of standard deviations its absorbance was above the MCAA‐negative control mean absorbance value.

### Antigen‐specific clearing of MCAA+ serum

A commercially available form of candidate antigen (human plasminogen; R&D Systems) was biotinylated in 360 *μ*L of 5 mmol/L Sulfo‐NHS‐SS biotin (Pierce) in PBS at 4°C for 2 h under constant rotation. Fifty microlitres of quenching solution was added to the reaction. The solution was cleaned of labeling reagent using a Pierce 10 kDa concentration unit at 14,000 × *g* and resuspended in 360 *μ*L of 25 mmol/L Tris buffered saline. The solution was then added to 250 *μ*L of neutra avidin bead suspension (Pierce) and kept under constant rotation for 1 h at 25°C. The column was centrifuged at 1000 × *g* for 2 min to remove excess solution. The column was washed twice with 500 *μ*L of 25 mmol/L tris buffered saline. Finally the column was capped, and loaded with 250 *μ*L of 25 mmol/L tris buffered saline and 105 *μ*L of MCAA+ serum. This was kept under constant rotation at 4°C for 12 h. The serum was collected from the column by uncapping the bottom and centrifuging at 1000 × *g* for 2 min. The serum was concentrated and washed two times with 500 *μ*L PBS by centrifugation in a 10 kDa concentrator. After the final wash, the serum was returned to the original concentration by adding enough PBS to bring the volume to 105 *μ*L.

### Cell‐based ELISA for detecting collagen

Human Met5a cells were plated into a 96‐well plate at 70,000 cells per well. The plates were incubated at 37°C for 3 h. The cells were then treated with pooled sera according to their treatment group designation which can be described as follows: No treatment, MCAA+, anti‐PLG, and MCAA+ serum cleared of anti‐PLG. The cells were then incubated for 82 h at 37°C and rinsed once with PBS. All blocking and incubation steps were performed under gentle agitation at room temperature for 1 h with washing steps occurring immediately following the incubation steps. The wells were then blocked with 200 *μ*L 5% nonfat dry milk in PBS. The wells were incubated with 2 *μ*g of mouse IgG targeted to collagen type 1 (ab6308; Abcam, Cambridge, MA) in 100 *μ*L of 3% BSA. The plates were then washed 3× with 200 *μ*L of 0.05% Tween in PBS for 4 min each wash. The plates were blocked again with 5% milk in PBS. The plates were then incubated with goat anti‐mouse IgG conjugated to HRP (Life Technologies). The plates were then washed 3× with 200 *μ*L of 0.05% Tween in PBS. The plates were developed with 100 *μ*L of Pierce One Step TMB ELISA reagent. The reactions were stopped with 50 *μ*L of 1 mol/L HCl. The plates were then read for absorbance at 450 nm on the BioTek plate reader.

### Statistical analysis

Two‐tailed, unpaired *t*‐tests and one way ANOVA tests were performed, using StatPlus (StatPlus Software, Walnut, CA). Fisher's exact tests were performed using GraphPad QuickCals online (http://graphpad.com/quickcalcs/contingency1.cfm). Statistical significance was defined as *P* < 0.05. Data are graphed with error bars indicating standard error of the mean (SEM). Graphs represent at least two experimental repetitions.

## Results

Following the affinity capture and elution of biotinylated surface proteins, the resulting protein mixture was separated into individual fractions by size exclusion chromatography. The resulting 143 fractions were analyzed for protein concentration, and then equalized. An equal amount of protein for each fraction was then used to detect MCAA binding in an ELISA. Binding by MCAA was statistically highest in samples 27 and 42 (*P* < 0.05) compared to all other samples (Fig. 1).

The MS/MS spectra obtained from an in‐solution digestion of fractions 27 and 42 was processed, using the Protein Lynx Global Server V2.3 (Waters Corp). The peak list files were analyzed via the MASCOT public database. It is important to note that fraction 30, which showed no MCAA binding (Fig. 1), was also analyzed.

To address the hypothesis that fractions which demonstrated MCAA binding contained proteins in common, the reports from MASCOT analysis for fractions 27 and 42 were compared. The lists were condensed to include only those proteins that had a match or near match in both reports. Any proteins found that were also present in fraction 30 (no MCAA binding) were considered to be unlikely candidates as key MCAA targets. The protein that had the highest MASCOT score of the remaining proteins was PLG (Table 2).

**Table 2:** Proteins of interest isolated via size exclusion chromatography identified by MS/MS

Location of fragments (Fraction #)	~Calculated/expected mass (Da)	Peptide sequence	Mascot Score 27, 30, 42	Proposed protein	NCBI accession no.	% sequence coverage
27, 42	1045.5291/1045.5342	K.LSSPADITDK.V	30, N/A, 34	Plasminogen	AAH60513	1%
27, 42	1045.5954/1045.5434 758.3923/758.3962	DATPLSR SYSTSLKSR	5, N/A, 20	Bromodomain PHD finger transcription factor	BAA89208	0.25%
27, 30, 42	1045.4676/1045.5342	ASEDGPLNSR	7, 17, 24	Interleukin‐25 isoform1 precursor	NP_073626	5%
27, 30, 42	1320.6708/1320.6778	STSGGTAALGCLVK	16, 19, 28	Immunoglobulin heavy chain variable region, partial	AAR32501	15%
27, 30, 42	1983.9902/1984.1533 855.5178/855.5050	ATSGTDTQYFGPGTRLTVL. AVVTTLPR	5, 17, 15	T‐cell receptor beta chain variable 20	BAF94857	5%

Digested fractions were examined on a Water's ESI‐QUAD‐TOF Mass spectrometer. Peaks lists were analyzed using MS/MS ion search. The Mascot score is a probability‐based MOWSE score. The ion score is −10 × log (*P*) where *P* can be understood as the probability that an observed match is a random event. Scores >22 for sample 27 and >38 for sample 42 indicate homology (*P* < 0.05). These scores are inferred from the size of the fragment being analyzed and ion scores observed in the second MS (McHugh and Arthur 2008). This truncated list includes only those proteins that were found in both fractions that showed MCAA binding in Figure 1.

**Figure 2:**
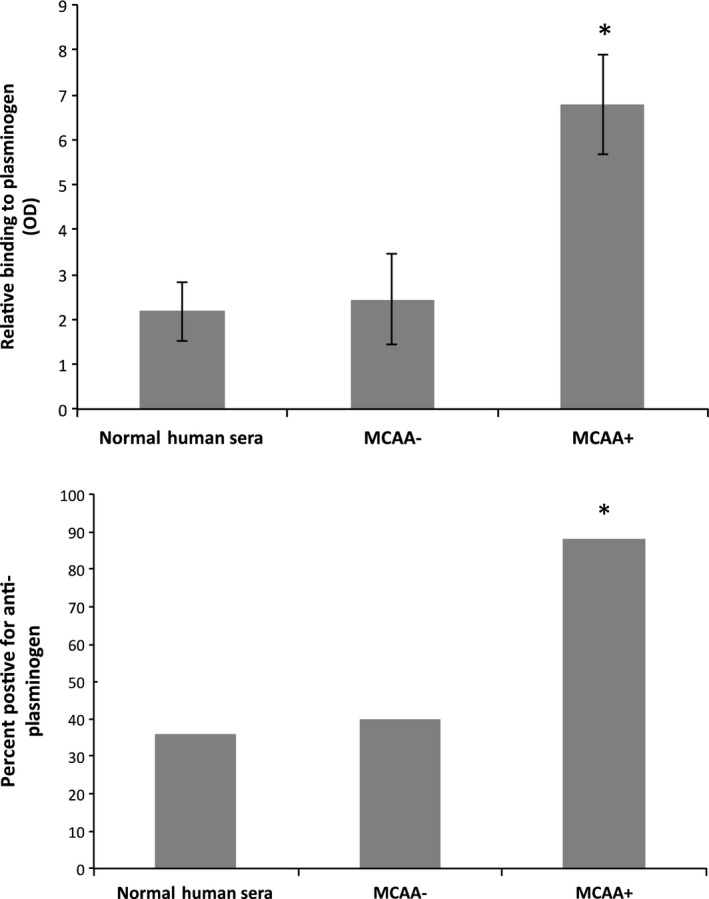
Binding of plasminogen by MCAA via ELISA. Plasminogen was coated in a 96 well plate and various treatment groups were added as described in the ELISA protocol. Normal Human Serum was obtained from Precision Med (Solana Beach, CA). The other two groups were sera taken from the population of Libby Montana, either positive or negative for MCAA. The plate was read at 450 nm. Top: Data indicate the average number of standard deviations above the mean absorbance for known negative control sera. *N* = 11–20 in each subset, *P < 0.01 by one‐way ANOVA and by two‐tailed *t*‐tests comparing to Normal Human Sera. Bottom: The percent positive for anti‐plasminogen among the subsets of serum samples. *N* = 11–20 in each subset, *P < 0.01 by Fisher's exact test.

The fact that a PLG homolog was identified in both fractions that demonstrated high binding from MCAA motivated us to test the binding of MCAA to PLG. The mean absorbance of binding by pooled MCAA+ serum was three times higher (*P* < 0.01) than the absorbance of MCAA‐negative samples or normal human serum (Fig. 2, top). In addition, 88% of the MCAA‐positive samples showed binding to PLG, suggesting that anti‐PLG antibodies are present in the majority of MCAA‐positive patients exposed to LA. This percentage was significantly higher than that seen in serum from MCAA‐negative patients or normal human serum (Fig. 2, bottom).

**Figure 3:**
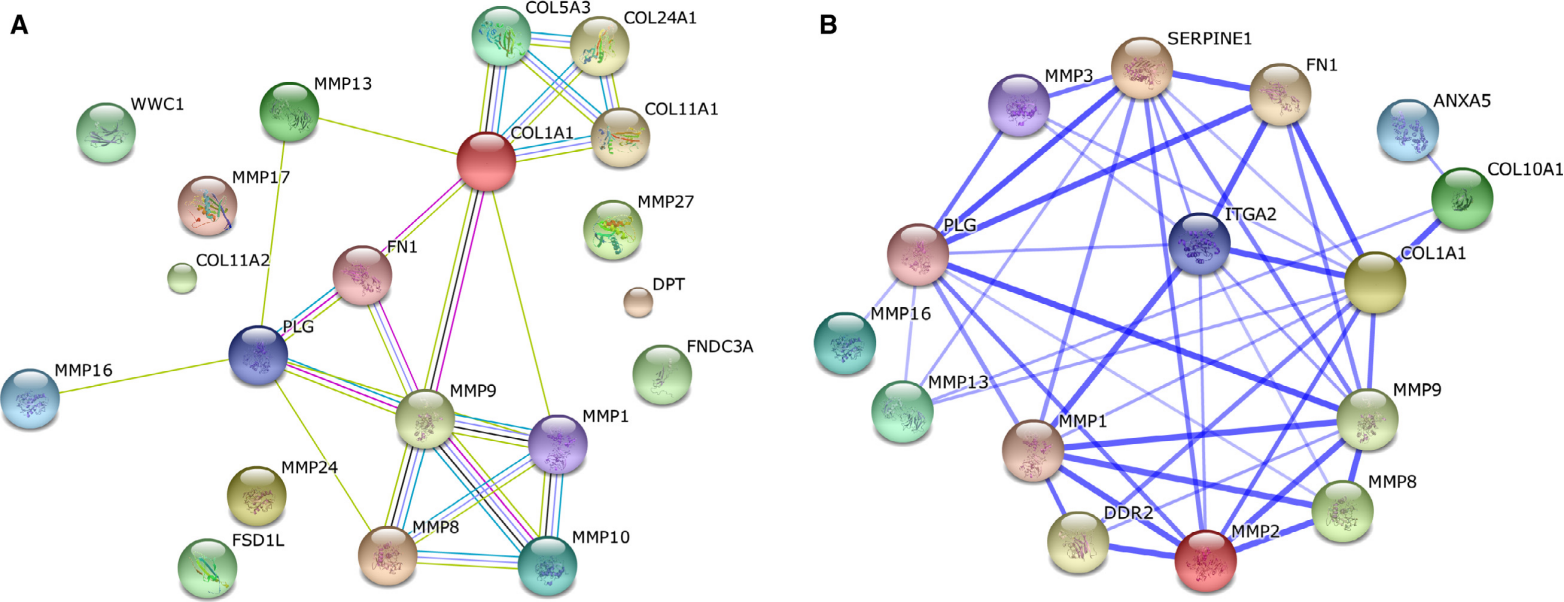
STRING summary networks. A brief search was performed using the STRING database to test the validity of the tandem mass spectrometry data. This search generated several networks that indicated proteins of interest that had functional relationships that could provide rationale for a mechanism by which PLG could affect COL1A1 expression. (A) Network view based on various comparison methods. Green correlates to genes located within 300 base pairs from each other on the genome; Dark blue relates genes by the frequency of occurrence together in various organisms; Black indicates instances of coexpression; Pink to standard molecular biology experiments; Sky blue to relationships detected in curated databases; Yellow shows relationships indicated by text‐mining of PubMed abstracts; and purple correlates to Homology. (B) Network view generated from a composite of all the scores evaluating the data supporting the connections observed in network A (Serve et al. 2013). Confidence scores were determined by calculating the probability of a functional interaction between two proteins based, off of the multiple lines of evidence. The method was validated against a baseline probability calculated from proteins with well‐known interactions from the KEGG database (Serve et al. 2013; Szklarczyk et al. 2015).

The searches performed using STRING 10 showed evidence that PLG interacts with at least nine proteins that also interact with COL1A1 in both *Homo sapiens* and *Mus musculus* (Jensen et al. 2009; Szklarczyk et al. 2015) (Fig. 3). Of these, the three with high confidence functional links to both PLG and Collagen were MMP‐9, MMP‐2, and ITGA2 (Tables 3 and 4). The most direct path with the highest score was between plasminogen and MMP9 with a score of 0.998. MMP9 connected to COL1A1 with a score of 0.811. Since MMP9 is a collagenase that is activated by PLG (Lund et al. 2011), this could serve as a possible mechanism for the collagen deposition observed in previous studies (Serve et al. 2013).

**Table 3:** Summary of STRING network

Protein symbol	Function of interest	Interaction with PLG score (HUM)	Interaction with Col1a1 score (HUM)	Interaction with PLG (MUS)	Interaction with Col1a1 score (MUS)
MMP9	Collagenase: breaks down Collagen type I, II, and III	0.998	0.811	0.923	0.638
MMP2	Collagenase	0.801	0.857	0.525	0.648
ITGA2	Integrin: interacts with collagen and up‐regulates genes that lead to modification of the ECM	0.586	0.985	0.629	0.481
MMP3	Collagenase	0.848	0.517	0.622	0
MMP1	Collagenase	0.632	0.549	0.622	0
MMP13	Collagenase	0.414	0.546	0	0.829
KRT18	Cytoskeletal keratin 18, surface receptor, implicated with moving mutated cystic fibrosis transmembrane receptor to the cell surface	0.64	0	0.254	0
DDR2	Receptor that interacts with collagen, upregulates expressions of MMP9	0	0.746	0	0
Annexin A5	Shields lipids that interact with coagulation processes	0	0	0	0

This table summarizes the strength of evidence for interactions between key proteins and plasminogen (PLG) and type 1 collagen (Col1a1) in both human (HUM) and murine databases. The interaction scores were determined by an algorithm that utilizes evidence based on genomic location, gene fusions, concurrence across genomes, co‐Expression, experimental/biochemical data, association in curated databases, and co‐mentions in Pub‐Med abstracts to calculate the final score. Scores >0.3 indicated medium confidence in the interaction between proteins while those with a score >0.7 were considered to be high confidence (Serve et al. 2013).

**Table 4:** Interactions of DDR2 and ITGA2

Interaction score with DDR2 (HUM)	
COL1A1	0.746
MMP9	0.574
MMP1	0.886
MMP2	0.993
Interaction score with ITGA2 (HUM)	
COL1A1	0.985
MMP9	0.581
MMP1	0.904
MMP2	0.56
MMP8	0.4

This table summarizes the strength of evidence for interactions between the collagen receptors DDR2 and ITGA2 and key genes within the network. The interaction scores were determined by an algorithm that utilizes evidence based on genomic location, gene fusions, concurrence across genomes, co‐expression, experimental/biochemical data, association in curated databases, and co‐mentions in Pub‐Med abstracts to calculate the final score (Serve et al. 2013).

**Figure 4:**
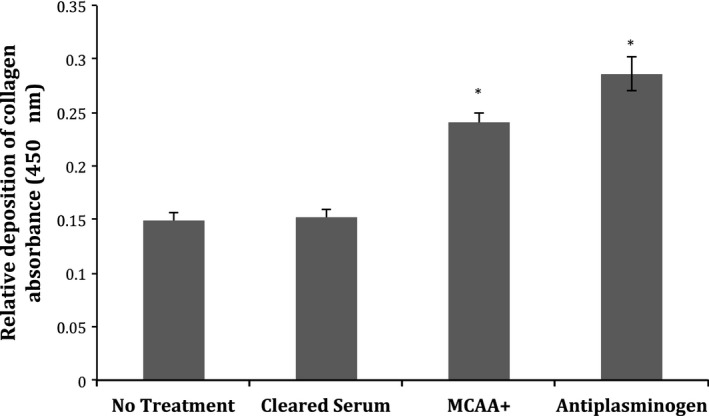
ELISA demonstrating the potential role of MCAA in collagen deposition. The collagen deposition induced by pooled MCAA+ serum was compared to the collagen deposition induced by a commercial antihuman plasminogen antibody. Error bars indicate SEM. Unpaired, two‐tailed *t*‐tests comparing the mean of treatment with No Treatment indicated that differences were statistically significant, *P < 0.05. *N* = 6 ELISA wells in each treatment group.

To determine whether PLG‐targeting MCAA had an activating or inhibiting effect on PLG, we compared the collagen deposition induced by a commercial anti‐plasminogen antibody that is known to inhibit PLG activity (R&D Systems, Monoclonal Mouse IgG_1_ Clone # 270409) to the deposition induced by exposure to MCAA. We found that both treatments increased collagen deposition higher than that of cells treated with cleared serum (Fig. 4). Statistical significance comparing treatments to cleared serum was determined via *t*‐tests with *P* values of 3.4E‐4 and 1.1E‐5, respectively.

**Figure 5:**
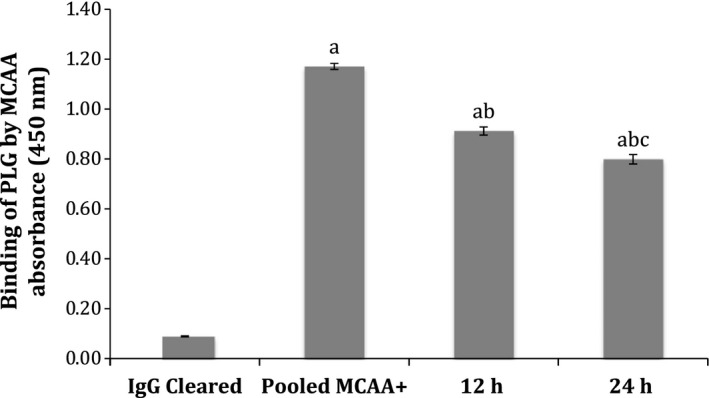
Binding of serum cleared of plasminogen‐targeting antibodies. Pooled serum that had been cleared of anti‐PLG antibodies was tested for MCAA+ binding to surface proteins on mesothelial cells. The binding of two samples that had been cleared for different times (12 and 24 h) were compared to binding of pooled serum cleared of all IgG (IgG Cleared) and un‐manipulated pooled MCAA+ serum. The binding of PLG‐cleared serum was higher than IgG cleared, but less than that of pooled MCAA+. The difference between groups was confirmed via unpaired, two‐tailed *t*‐test with *P*‐values <0.05 when compared with IgG Cleared (a), compared with pooled MCAA (b), and 12 h versus 24 h (c). *N* = 6 wells in each treatment group.

To discover whether or not other proteins were being targeted by MCAA, we compared binding of unmanipulated pooled MCAA+ serum to Met5a cells with binding observed by (1) pooled serum that had been cleared of PLG targeting antibodies and (2) pooled serum that had been cleared of all IgG. The results of this cell‐based ELISA showed that the binding of serum cleared of PLG targeting antibodies was statistically lower than whole MCAA+ serum (*P* = 3.6E‐7) and statistically higher than serum cleared of all IgG (*P* = 4.1E‐8) (Fig. 5). However, because binding did not drop all the way to control levels when serum was cleared of anti‐PLG antibodies, the data indicate that other subsets of MCAA exist in the sera that target different proteins on the Met5a cell.

**Figure 6:**
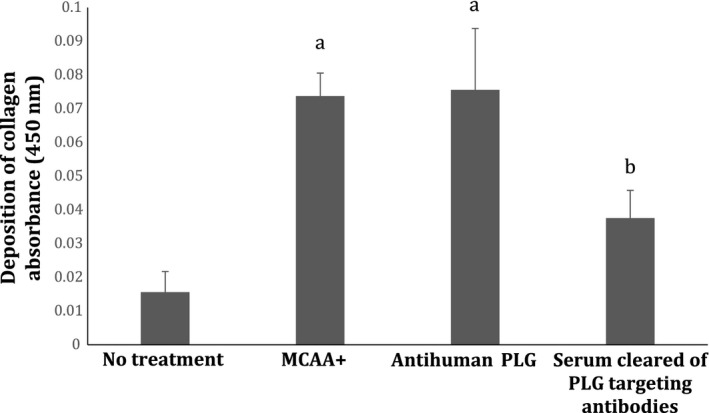
Collagen deposition by cells treated with serum cleared of PLG targeting MCAA. To test how much of the collagen deposition induced by MCAA could be attributed to the presence of the PLG‐targeting MCAA subset, the collagen deposition induced by pooled MCAA+ serum was compared to that induced by pooled MCAA+ serum that had been cleared of anti‐PLG antibodies. The observed difference between MCCA+ and No treatment was statistically significant when analyzed via unpaired, two‐tailed *t*‐test *P* < 0.05 (a). Clearing the anti‐PLG antibodies using affinity chromatography significantly reduced collagen production, *t*‐test *P* < 0.05 compared to MCAA treatment (b). *N* = 6 ELISA wells in each treatment group.

To approximate how much of the induced collagen deposition could be accounted for by the PLG‐targeting MCAA, we compared the collagen deposition induced by pooled whole MCAA+ serum to that induced by pooled serum that had been cleared only of PLG‐targeting MCAA antibodies (Fig. 6). We found that the collagen deposition induced by MCAA+ serum was significantly reduced (*P* = 0.001), virtually to control levels, when the anti‐PLG antibodies were cleared from the serum.

## Discussion

Exposure to LA asbestos drives production of various autoantibodies as well as a progressive form of pleural fibrosis termed LPT (Pfau et al. 2005, 2015; Marchand et al. 2012; Black et al. 2014). The potential for autoantibodies to exacerbate fibrotic disorders has been suggested by other studies (Chizzolini et al. 2002; Baroni et al. 2006; Pfau et al. 2011). In those cases, primarily in systemic sclerosis, the fibrosis occurred in the lung and the mechanism was hypothesized to be binding of anti‐fibroblast antibodies (AFA) to surface receptors such as Platelet‐Derived Growth Factor Receptors (PDGF‐R) that play a role in collagen deposition. However, for pleural fibrosis, the primary disease outcome from LA exposure, it seemed more likely that the autoantibodies would target mesothelial cells that line the pleural cavity. In support of this hypothesis, in the case of the Libby LPT, we have previously demonstrated the presence of autoantibodies to mesothelial cells (MCAA) in LA‐exposed subjects, as well as an association between the presence of MCAA and pleural fibrosis (Marchand et al. 2012). However, the mechanism whereby these autoantibodies would induce or exacerbate pleural fibrosis is unknown.

In order to begin to understand that mechanism, this study was designed to use proteomics approaches to identify potential cell surface targets for the MCAA. Surface proteins were collected by biotinylating the extracellular domains on whole cells, and then extracting from the whole cell lysates via an avidin affinity column. The resulting proteins were separated into size fractions, which were probed for binding using an ELISA format. High binding fractions were then analyzed by MS/MS.

Fractions 27 and 42 from the size exclusion separation were selected for MS/MS analysis because they displayed the highest absorbance when probed for MCAA binding. This indicated that proteins with an epitope for MCAA made up a larger percentage of the total protein in these fractions, compared with other fractions. Therefore, the MS/MS profiles of the proteins in these fractions would provide higher quality data for protein inference.

The top fifty hits of each MASCOT analysis were examined. Of those highest‐scoring proteins reported from each MS/MS analysis, only five proteins identified in sample 42 had exact or near matches in sample 27 (Table 2). Of these, three were determined unsuitable as they had exact matches in sample 30, which demonstrated low binding by MCAA+ sera. Nothing in the literature indicated that the bromodomain PHD finger transcription factor had direct ties to the expression of collagen. This left plasminogen as the most likely target for MCAA.

A review of the literature revealed a precedent with regard to the role of plasminogen in fibrosis. Activation of the plasminogen/plasmin system has been shown to slow progression of fibrosis in experimentally induced asbestos disease for murine models (Hattori et al. 2004; Obi et al. 2014). Deficiencies in plasmin or plasminogen activator have led to increased fibrosis (de Giorgio‐Miller et al. 2005). Therefore, autoantibody binding that inhibited this system would be expected to increase fibrotic collagen deposition. It will therefore be important in future studies to evaluate the effects of the presence of anti‐PLG on plasmin levels in patients.

The next logical step was to test for binding of the MCAA to PLG to demonstrate the presence of anti‐PLG autoantibodies in the sera from LA‐exposed subjects. The mean absorbance from the binding ELISA using commercial purified PLG was three times that of the control groups as seen in Figure 2. This confirmed that PLG is the most likely candidate for our target protein isolated from the size exclusion method.

The STRING 10 protein interaction network was selected to analyze potential connections to ECM deposition or degradation because it provides weighted connections within the generated networks (von Mering et al. 2005; Szklarczyk et al. 2015). When connections between plasminogen/plasmin system and collagen type 1 were queried, using the STRING database, multiple two‐node pathways were found between plasminogen and Collagen type 1 in both *Homo sapiens* and *Mus musculus*. The most noteworthy connection was between plasminogen, MMP9, and Collagen Type 1. Since MMP9 is a collagenase that is activated by plasminogen (Lund et al. 2011), this could provide a possible mechanism for the collagen deposition observed in previous studies (Serve et al. 2013).

It is interesting to note that two of the tyrosine receptor kinases (TRK) found in our search interact with collagen and contribute to governing ECM composition (Table 4). Discoidin domain containing receptor 2 (DDR2) and ITGA2 both assist in collagen regulation and are implicated in the upregulation of matrix metalloproteases (Riikonen et al. 1995; Poudel et al. 2015). It is possible that an imbalance in the degradation of collagen would result in an upregulation of these pathways to increase production of MMPs in their inactive form. While there is no evidence yet supporting a role for DDR2 in pleural fibrosis, involvement in lung fibrosis has been demonstrated and a loss of DDR2 results in promotion of hepatic fibrosis (Olaso et al. 2011; Yang et al. 2013).

We also felt that it was important to address three other questions regarding the involvement of PLG in the excess collagen deposition observed: (1) Whether MCAA was activating or deactivating PLG; (2) whether PLG was the only protein being targeted by MCAA; and (3) approximately how much of the excess collagen deposition could be attributed to the binding of PLG by MCAA.

To investigate if MCAA binding was activating or deactivating plasminogen, we exposed Met5a cells to a commercial antihuman plasminogen antibody that inhibits plasminogen activity. Although the commercial anti‐PLG was a mouse monoclonal antibody, so quite different in isotype and epitope specificities, its usefulness was its known inhibitory activity. This allowed us to compare the functional effects of the MCAA in the sera, which we have shown to contain human anti‐PLG. Collagen Type 1 deposition was upregulated by both the commercial anti‐PLG antibody and MCAA, compared to that of cleared serum, suggesting that the anti‐PLG in MCAA inhibits its activity. This is consistent with a potential role for PLG in fibrosis, since deficiencies in plasmin or plasminogen activator have led to increased fibrosis (de Giorgio‐Miller et al. 2005).

To test whether PLG was the only protein on Met5a cells targeted by MCAA, we performed a cell‐based ELISA which compared the binding of whole MCAA+ serum to that of serum that had been cleared of PLG targeting antibodies. The results demonstrated that the binding of serum cleared of PLG targeting antibodies was less than that of whole serum (*P* = 0.001) but greater than binding of serum cleared of all IgG (*P* = 4.1E‐8). This indicates that there are other cell surface proteins being targeted by other subsets of MCAA.

Our final experiment was designed to test the most intriguing finding from our MS/MS procedure. We wanted to determine whether or not any of the collagen deposition induced by MCAA+ serum could be attributed to the subset of PLG targeting antibodies. To do this, we compared the collagen deposition induced by whole MCAA+ serum to that of serum that had been cleared of PLG targeting antibodies. Simply using an affinity column to remove antibodies to human plasminogen allowed us to use the serum with minimal manipulation, where the only component missing from the serum treatment was the anti‐PLG. Our results showed that the collagen deposition was significantly reduced (*P* = 0.001) when anti‐PLG antibodies were cleared from the serum. This suggests that PLG is a target of MCAA and that this interaction has the ability to contribute to increased collagen deposition in Met5a cells as observed by Serve (Serve et al. 2013).

One limitation of this study occurred because we were unable to analyze all of the fractions. Because of this, it was impossible validate the efficiency of our recovery of plasma membrane protein. Though it was evident that we had successfully extracted membrane surface proteins, it was likely that the extraction did not obtain all surface proteins. As a consequence, some potential targets may not have been liberated from the plasma membrane and would not have been detected via the binding of MCCA. The data from Figure 5 clearly show that the LA‐exposed serum samples contain MCAA that target proteins other than plasminogen. Further studies are warranted to identify other potential targets.

## Future work

The first step to further this work could be to confirm the role of the plasminogen–plasmin system in the excess collagen deposition in vivo, in a murine model. Since maintenance of the ECM involves many regulatory systems, it is important to confirm that the manipulation of one system is enough to lead to the observed symptoms. Our previous work demonstrated that the collagen deposition by Met5A cells induced by MCAA does not require expression of smooth muscle alpha actin or epithelial‐mesenchymal transition (EMT) (Serve et al. 2013), suggesting a different pathway. Our STRING search connections provide hypotheses for studies on molecular mechanisms involved. Other work will also be needed to identify other possible MCAA targets that may have been missed as discussed above. Further, it will be important to examine the impact of anti‐PLG on disease outcomes in the Libby population. This will be critical in the development of therapeutic strategies based off of a true understanding of the pathogenesis of LPT.

## Conclusion

We were able to show that a subset of MCAA preferentially bind plasminogen. We have also shown that it is likely that MCAA binding to plasminogen contributes to a sizable portion of the excess collagen deposition seen in Met5a cells exposed to asbestos‐induced MCAA. The similarity of this effect with the outcome of an inhibitory commercial anti‐PLG suggests that the MCAA that target plasminogen inhibit its activity. These results provide strong evidence for a novel mechanism of pathogenesis by MCAA. It also provides a good foundation for identifying possible therapeutic targets for those suffering from LPT.

## Conflict of Interest

None declared.
